# Spatial variability of climate effects on ischemic heart disease hospitalization rates for the period 1989-2006 in Quebec, Canada

**DOI:** 10.1186/1476-072X-9-5

**Published:** 2010-02-08

**Authors:** Lampouguin Bayentin, Salaheddine El Adlouni, Taha BMJ Ouarda, Pierre Gosselin, Bernard Doyon, Fateh Chebana

**Affiliations:** 1 Institut National de la Recherche Scientifique - Eau, Terre et Environnement (INRS-ETE), 490 de la Couronne, Québec, QC G1K 9A9, Canada; 2 Institut National de Statistique et d'Économie Appliquée (INSEA), B.P: 6217, Madinat Al Irfane, Rabat-Instituts, 10100 - Rabat, Morocco; 3 Institut National de Santé Publique du Québec (INSPQ) and Université Laval, 945, avenue Wolfe, Québec, QC G1V 5B3, Canada

## Abstract

**Background:**

Studies have suggested an association between climate variables and circulatory diseases. The short-term effect of climate conditions on the incidence of ischemic heart disease (IHD) over the 1989-2006 period was examined for Quebec's 18 health regions.

**Methods:**

Analyses were carried out for two age groups. A GAM statistical model, that blends the properties of generalized linear models with additive models, was used to fit the standardized daily hospitalization rates for IHD and their relationship with climatic conditions up to two weeks prior to the day of admission, controlling for time trends, day of the season and gender.

**Results:**

Results show that, in most of Quebec's regions, cold temperatures during winter months and hot episodes during the summer months are associated with an increase of up to 12% in the daily hospital admission rate for IHD but also show decreased risks in some areas. The risk of hospitalization is higher for men and women of 45-64 years and varies spatially. In most regions, exposure to a continuous period of cold or hot temperature was more harmful than just one isolated day of extreme weather. Men aged 45-64 years showed higher risk levels of IHD than women of the same age group. In most regions, the annual maximum of daily IHD admissions for 65 years old was reached earlier in the season for both genders and both seasons compared to younger age groups. The effects of meteorological variables on the daily IHD admissions rate were more pronounced in regions with high smoking prevalence and high deprivation index.

**Conclusion:**

This study highlights the differential effects of cold and hot periods on IHD in Quebec health regions depending on age, sex, and other factors such as smoking, behaviour and deprivation levels.

## Background

A decline in the rates of deaths and of hospital admissions is reported for cardiovascular diseases in Canada [1], while most risk factors, except smoking have been rising simultaneously throughout the country [2,3]. Aside from known risk factors, climate variables, especially temperature and relative humidity, are documented to have an association with circulatory diseases [4-6]. The overall mean temperature as well as extreme weather events across the planet are projected to increase, according to future climate simulations [7]. Therefore, the definition of meteorological variables effects on the population health is regarded as important in the governmental policy making process.

In Quebec, a study has confirmed a significant association between the historic temperature and mortality [8]. Most studies have so far focused on mortality and reported a non-linear relation (U, J, or V shaped) between daily mortality and extreme temperatures. The few studies that focused on morbidity reported an increase in daily hospitalizations for cardiovascular and respiratory diseases during extreme weather conditions [5,9-11]. It has also been reported that temperature effects are observed up to a few days after initial exposure [12].

In this study we investigate the impact of meteorological variables on the incidence of ischemic heart diseases (IHD, ICD9:410-414.9) in the province of Quebec (Canada) over the period 1989-2006. The main objective is to estimate the effects of climate variables on IHD hospital admissions while taking into account as much as possible confounding variables or risk factors such as gender, age group, material and social deprivation, smoking and the geographic health region. This represents a first step towards establishing future projections for hospital admissions under climate change scenarios.

## Methods

### Health and related data

**Figure 1:**
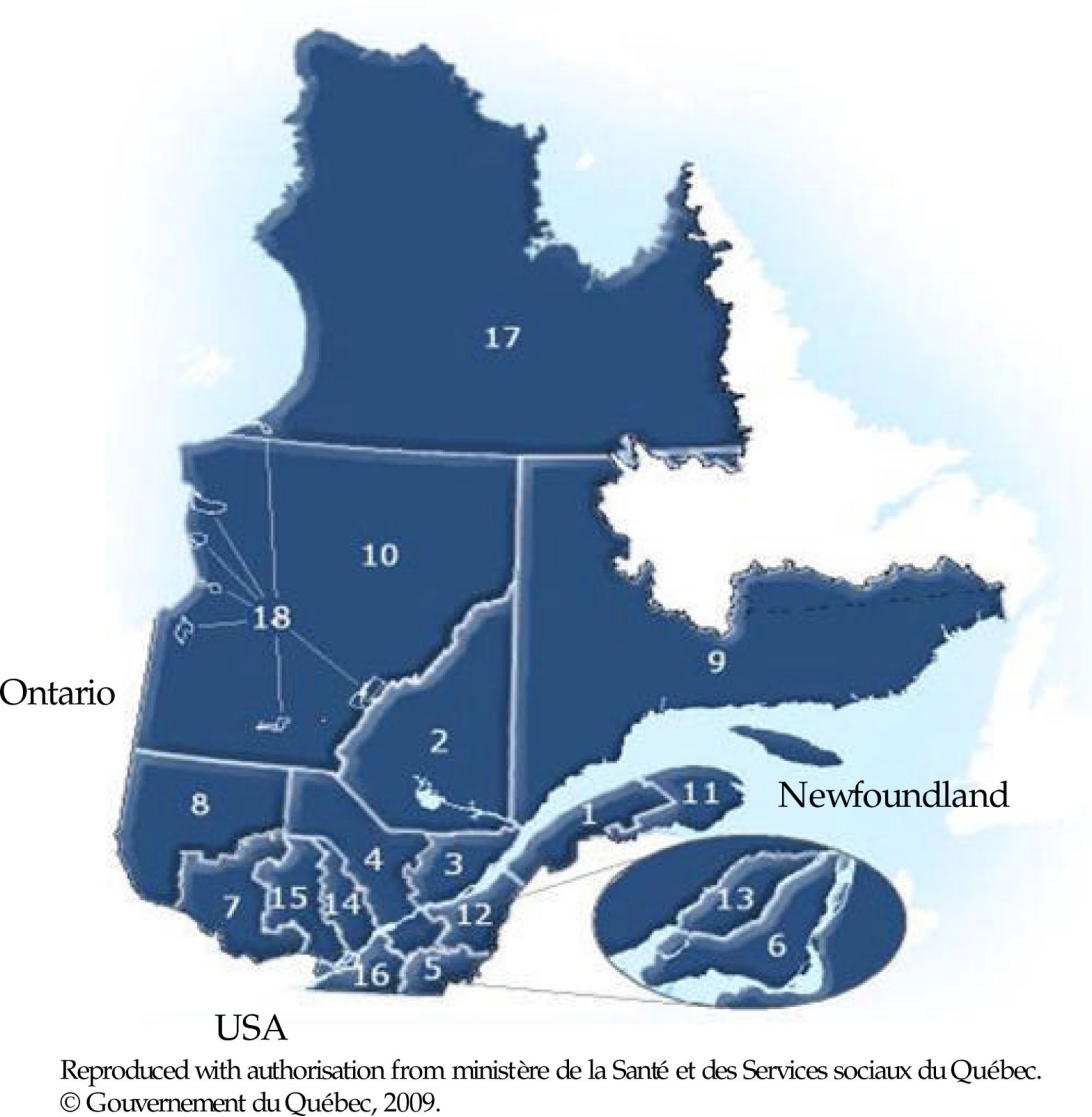
**Health regions limits of the province of Québec**. Health region-Name (population density in inhabitants per km^2^). 01-Bas Saint Laurent (9.1); 02-Saguenay Lac Saint-Jean (2.9); 03-Capitale Nationale (36.5); 04-Mauricie et Centre du Québec (11.61); 05-Estrie (29.9); 06-Montreal (3769.1); 07-Outaouais (11.5); 08-Abitibi-Témiscamingue (2.5); 09-Côte-Nord (0.4); 10-Nord-du-Québec (0.1); 11-Gaspésie-Îles-de-la-Madeleine (4.7); 12-Chaudière-Appalaches (26.7); 13-Laval (1562.0); 14-Lanaudière (36.8); 15-Laurentides (26.0); 16-Montérégie (127.4); 17-Nunavik (0.02); 18-Terres-Cries-de-la-Baie-James (2.28).

Daily hospitalizations for IHD, all ages, from April 1^st ^1989 to March 31^st ^2006 were extracted from the hospital discharge database (Med-Echo) supplied by the *Ministère de la santé et des services sociaux du Québec *(MSSS). With the estimated annual population data, a direct standardization [13] was conducted for age and gender to obtain a time series of the daily IHD admissions rate for each one of Quebec health regions (Figure 1). The three Northern regions (10, 17 and 18, located north of the 50^th ^parallel) were excluded from the study due to a lack of homogeneous data

Data on smoking prevalence by age group and by sex for the periods 1998, 2000-2001 and 2003 were obtained from the *Institut national de santé publique du Québec *(INSPQ); no comparable data was available for the period 1989-1997. Data on the deprivation index was also provided by INSPQ. This index has two dimensions: material and social [14-16] and is available at the dissemination area level [17] or its former equivalent, every five years, since 1996. Again no comparable data was available for the period 1989-1995. The indicators for other risk factors (e.g. obesity, diabetes, and hypertension) were unavailable at the spatial scale of interest for this study All preceding indicators were thus not included in the model and they were used for discussion purposes only.

### Meteorological data

Observed meteorological data come from Environment Canada's National Climate Archives [18]. The same data were used for the regions of Laval and Montreal because of their proximity. Table 1 gives a summary of available meteorological data. December, January and February are considered as the winter months while June, July and August are the summer months. Due to missing meteorological data for the regions 04, 07, 12 and 14, the choice of variables was limited to daily mean temperatures, precipitations and ground snow.

**Table 1:** Meteorological variables

Variable	Unit
Daily mean temperature	°C

Relative humidity	%

Precipitations	mm

Atmospheric pressure	Pa

Dew point	°C

Ground snow	mm

### Statistical analysis

A generalized additive model (GAM) [19] was used to model climate effects on IDH hospital admissions. Parameter estimation is carried out with the approach combining smoothing parameter λ with the B-splines basis function [20]. The variables to be included in the model are selected by a forward stepwise selection technique and an F-test is conducted in the comparison of two different models [19]. Prior to variables selection, a lag search is performed using the generalized cross-validation (*GCV*) criterion. For each variable, the mean of past observations up to two weeks is computed and the number of days providing the smallest model *GCV *is added to the list of covariables. The parameter λ, is estimated by comparing the Akaïke information criterion (*AIC*) values corresponding to different values of λ (λ = 0.001, 5, 15, 30, 60, 90 and 180). The excess risk is the relative change in the daily rate of IHD admission due to a decrease of 1°C below a temperature threshold for winter and an increase of 1°C above a threshold in summer.

## Results

### Descriptive statistics

**Figure 2:**
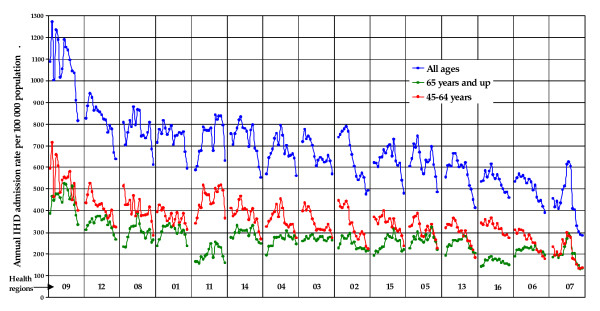
**Men's annual IHD hospital admission rate by health region (1990-2006)**. The regions are ranked from the highest to the lowest annual mean admission. For each region, the data represent annual admission rates for IHD for consecutive time periods from 1990 to 2006.

**Figure 3:**
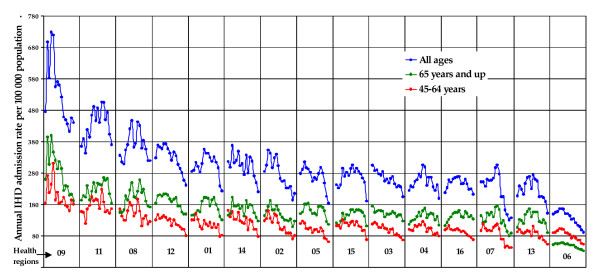
**Women's annual IHD hospital admission rate by health region (1990-2006)**. The regions are ranked from the highest to the lowest annual mean admission. For each region, the data represent annual admission rates for IHD for consecutive time periods from 1990 to 2006.

**Figure 4:**
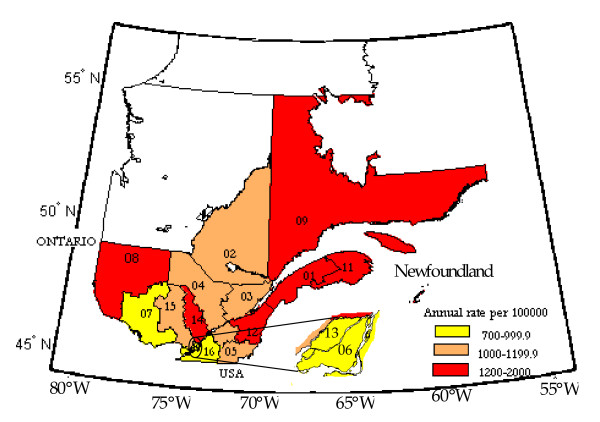
**Annual IHD standardized hospital admission rates for the age group of 45 years and up and both genders**.

Most cases of IHD hospital admissions were in the age group 45-64 for men and 65 and over for women. Annual IHD admission rates are illustrated in Figure 2 for men and Figure 3 for women. The annual admission rate showed an increasing trend in all regions and age groups for both genders in the first half of the nineties. By the beginning of 2000 the admission rate was decreasing steadily in all regions. Higher annual IHD admission rates for men and women of 45 years and more are observed mostly in the North-Eastern regions and the lowest rates are in the South-Western regions (Figure 4). Overall, the data shows a decreasing rate from North to South and from East to West.

### Models for winter

**Figure 5:**
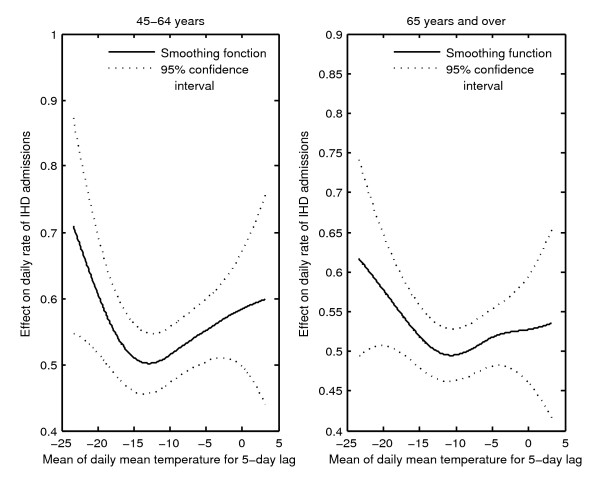
**Effect of averaged daily mean temperatures for a 5-day lag on daily rate of IHD hospital admissions for men in the health region of Capitale-Nationale in winter (typical curve)**.

**Figure 6:**
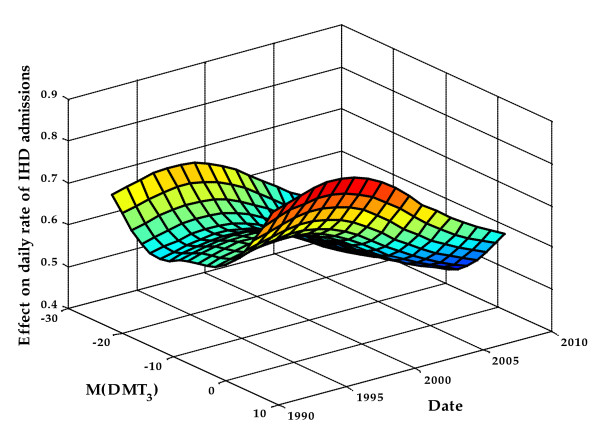
**Effect of averaged daily mean temperatures for a 3-day lag on daily rate of IHD hospital admissions over time, of 45-64 years old men in the health region of the Montreal in winter**. M(DMT_3_) = averaged daily mean temperatures for a 3-day lag period.

In most cases, delayed variables (i.e. variables with a lag) were the first meteorological variables to be selected for the model. This indicates that prior climatic conditions have more impact on IHD hospital admission than those of the day of admission. For both genders and in most regions, IHD daily admission rates were increasing with averaged daily mean temperatures for a given lag and with same-day mean temperature. The widely reported V or U-shaped curves obtained through the GAM model best described this association (Figure 5). For men, a stronger effect on IHD daily hospital admissions was observed in the 45-64 years age group, whereas for women there was no particular dominance of any age group despite the fact that the IHD admission rate was higher in the 65 years and over group. The V or U-shape was also observed with humidity and dew point. The daily IHD admission rate also increased with precipitation and ground snow for both genders. Furthermore the effects of meteorological variables were decreasing over time during the period under study (Figure 6).

Table 2 presents the excess risk of the daily rate of IHD admission for men and women related to a decrease of 1°C below a threshold. The threshold was defined as the inflexion point of the smoothing function and was different for each region and for both genders. In most regions, the effect of a 1°C drop in the averaged daily mean temperatures for a given lag is stronger than the one corresponding to a same-day mean temperature drop. This means that an exposure to continuous cold temperatures is more potent for IHD hospital admissions. Note that the duration of the lag depends on the variable and the region.

**Table 2:** Excess risk (%) due to a 1°C drop below a threshold in winter

Sexe	Men				Women			
**Age**	**45-64 years**		**65 and up**		**45-64 years**		**65 and up**	

**HR^+^**	M(DMT_L_)*	**DMT**	**M(DMT_L_)**	**DMT**	M(DMT_L_)*	**DMT**	**M(DMT_L_)**	**DMT**

01	**12.32**	**-9.90**	**-1.59**	-1.28	**1.52**	-0.03	**1.20**	**0.34**

02	-3.21	**0.65**	**-11.65**	-1.83	**-2.27**	-0.01	**-1.70**	-0.10

03	**1.72**	**0.95**	**1.07**	0.06	0.00	**-4.55**	**-1.66**	**0.20**

04	-0.48	**1.32**	0.81	0.09	-0.15	**-7.20**	**-0.79**	**0.73**

05	7.73	-4.17	-2.69	-2.74	**4.46**	-0.10	**-3.59**	-0.02

06	**1.03**	0.56	**0.53**	**0.57**	**-1.84**	0.11	-0.49	-0.01

07	**1.51**	**1.05**	**1.31**	**1.12**	**2.33**	**0.65**	-0.71	**2.88**

08	2.42	-0.12	2.79	1.72	**7.67**	**-1.80**	na♦	na

09	7.55	-1.63	-7.44	0.42	6.69	**11.88**	**14.88**	**-11.35**

11	6.42	0.91	3.18	**2.98**	2.07	-0.41	-0.07	**-3.24**

12	-1.57	1.04	2.03	0.13	**-6.52**	**-4.13**	**-1.09**	0.30

13	-0.91	**2.11**	-1.20	0.28	-0.48	**-0.78**	**1.21**	-0.09

14	**7.22**	-0.29	1.70	**-2.22**	**-7.36**	**-1.07**	**-0.93**	0.26

15	-0.72	-0.55	**-2.36**	0.53	**1.60**	**-1.10**	**1.47**	**0.81**

16	0.60	**0.13**	**0.57**	**0.79**	**-1.28**	**-0.25**	0.07	**-0.55**

* DMT = daily mean temperature,

M(DMT_L_) = averaged daily mean temperatures for a given lag

♦ na: not available

^+ ^HR stands for health region

In bold are values significantly different from zero (α = 0.01)

For men, the risk was higher (1.03% to 12.32%) in the 45-64 years age group in most regions, compared to older men (0.53% to 2.98%). There was a protective effect (decreasing risk) of cold temperature on IHD admissions rate in few regions.

The highest excess risks for women were in region Côte-Nord for both age groups. The protective effect of cold was more common for women across the province. Note that for both genders, the regions with the highest excess risk were in the group with the highest IHD annual rate. The low annual rate group also presented the lowest excess risk for men.

Maximum daily rates for men were observed in January or February. For most regions January was the high risk period whereas in a few, it was late February. Regions with February peaks were North-Eastern regions and North-Western regions. Peaks in hospital admissions were observed earlier in the season for men 65 years and over when compared to the 45-64 years age group. For women, maximum rates were observed at the end of December and early January. High rates were also observed earlier for 65 years old and up women compared to younger ones.

### Models for summer

**Figure 7:**
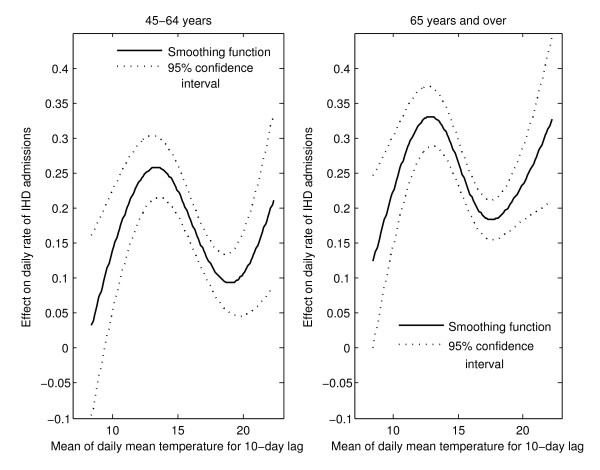
**Effect of averaged daily mean temperatures for a 10-day lag period on daily rate of IHD hospital admissions for women in the health region of Bas-Saint-Laurent in summer (typical curve)**.

The association with averaged daily mean temperatures for a given lag was typically in the shape shown in Figure 7, instead of the commonly reported U or J form. There are two increases at both extremes of the hot temperature range separated by a decrease in daily rate of IHD admissions, approximately between the temperatures of 14°C and 18°C. That shape was observed for both genders in most regions. Women aged 45-65 years were more affected by extreme heat in comparison to men of the same age (Table 3). Other than this general statement, no specific pattern was discernible.

**Table 3:** Excess risk (%) due to a 1°C rise above a threshold in summer

Sexe	Men				Women			
**Age**	**45-64 years**		**65 and up**		**45-64 years**		**65 and up**	

**HR^+^**	M(DMT_L_)*	**DMT**	**M(DMT_L_)**	**DMT**	M(DMT_L_)*	**DMT**	**M(DMT_L_)**	**DMT**

01	0.71	0.71	5.99	1.23	2.75	**6.59**	**3.47**	**-1.78**

02	**-4.25**	0.61	**-11.56**	**-2.66**	**-4.24**	**-4.60**	**-4.52**	**-6.05**

03	1.06	0.26	**-5.06**	0.26	**-4.87**	**-1.65**	**-1.72**	-0.80

04	-0.30	0.65	1.84	0.33	**1.36**	**-4.93**	**-3.12**	**-10.90**

05	**2.53**	0.87	0.22	-0.66	**5.04**	**2.86**	-0.64	**-15.39**

06	-0.72	0.52	-0.43	**1.08**	**3.08**	**0.82**	**3.06**	**1.55**

07	-1.89	**-2.32**	-12.03	0.29	**10.07**	**6.76**	**-18.39**	**-19.02**

08	5.80	4.19	**4.15**	**5.48**	**-13.35**	0.87	**-6.80**	**3.17**

09	1.77	15.58	**-13.62**	11.53	**6.50**	0.97	4.12	**2.76**

11	-2.58	-1.63	0.73	-0.39	**3.47**	**-8.53**	**2.41**	**-5.75**

12	1.39	1.46	-0.79	0.84	0.38	**6.39**	0.26	**4.02**

13	**-9.96**	**8.13**	-0.26	-2.49	1.61	**-6.95**	**-3.67**	**-4.60**

14	**-4.38**	0.54	**-2.87**	0.99	**5.81**	**-3.48**	**13.08**	0.72

15	**-8.99**	**2.77**	na^♦^	na	**-4.12**	**4.28**	0.44	-0.30

16	2.14	-0.39	**1.58**	**2.13**	**1.21**	-0.12	0.08	0.72

* DMT = daily mean temperature; M(DMT_L_) = averaged daily mean temperatures for a given lag; ♦ na: not available.

^+ ^HR stands for health region

In bold are values significantly different from zero (α = 0.01)

During summer, for both genders, higher values of the daily admission rate were recorded earlier in the season for the 65 years and over age group in comparison to the 45-64 years age group. Most regions, especially regions with large cities, presented a decrease in 65 years old men's daily admission rate around mid-July, while an increase is observed in the 45-64 years old men's rate.

### Risk factors and health determinants

Besides age, sex and health region, no risk factor was incorporated into the models, given major data limitations. To investigate the differences in weather effects amongst health regions, the mean smoking prevalence was checked for the province of Quebec (Table 4). The smoking prevalence for the age group of 45-64 years was twice that of 65 years and over, for both genders. For the purpose of this study the smoking prevalence was categorized into three groups: high (superior to 27.7%), moderate (between 16.5% and 27.7%) and low (inferior to 16.5%). The smoking prevalence for men aged 45-64 years old is high in almost all regions and is either high or moderate in all regions for women of the same age. The smoking prevalence for men 65 years and over was moderate in most regions; while older women generally showed a low prevalence. The comparison of weather-related excess risks and smoking prevalence showed that for both seasons and for men and women of the 45-64 years age group, the regions that have statistically significant high excess risk also presented high smoking prevalence. Note that some regions with moderate or low smoking prevalence also displayed significant excess risk for the 65 years and over age group.

**Table 4:** Men and women smoking prevalence mean (%) by age group and health region (1998, 2000-2001, 2003)*

	Men		Women	
**HR^+^**	**45-64 years**	**65 and up**	**45-64 years**	**65 and up**

01	25.7	18.7	24.6	5.3

02	32.2	14.8	22.0	12.7

03	31.9	14.5	24.3	13.3

04	33.3	21.7	26.3	9.1

05	32.1	12.6	25.1	10.5

06	32.7	15.4	28.9	15.3

07	34.8	23.9	31.3	17.3

08	29.3	18.5	28.2	12.2

09	30.7	16.7	30.2	11.5

11	28.1	16.8	26.8	10.2

12	29.8	18.3	21.2	9.9

13	30.9	11.8	30.2	14.6

14	38.7	22.2	35.1	15.0

15	32.0	18.1	29.0	14.5

16	26.8	13.5	29.5	12.0

* Data for 2003 were not available for all regions for the age group of 65 years and over.

^+ ^HR stands for health region

Another important factor taken into account is the deprivation indexes for the province [15]. Highest values of material deprivation (or the most deprived materially) are in North-Eastern regions such as Gaspésie-Îles-de-la-Madeleine (Table 5) and the highest values of social deprivation (or the most deprived socially) are in metropolitan regions such as Montreal (Table 5). Those regions displayed significant excess risks either for winter or summer models.

**Table 5:** Percentage of the region's population that scores in the highest quintile of material and social deprivation indexes* (2001)

	i^th ^quintile for material dimension					i^th ^quintile for social dimension				
**HR^+^**	**1**	**2**	**3**	**4**	**5**	**1**	**2**	**3**	**4**	**5**

01	3.9	15.6	16.4	20.2	43.9	25.6	25.4	26.4	17.5	5.1

02	3.8	15.0	20.2	27.4	33.6	28.7	26.0	18.9	17.3	9.1

03	30.7	22.2	18.3	15.9	13.0	17.0	21.4	18.4	18.2	25

04	7.85	14.8	24.2	28.55	24.55	17.6	24.7	23.6	17.2	17

05	11.5	26.5	22	24.3	15.6	16.7	21.8	22.2	19.2	20

06	28.2	18.5	16.4	17.0	19.9	12.1	10.5	14.5	26.6	36.3

07	30.0	17.4	14.5	16.2	21.8	22.3	19.1	18.1	19.4	21.1

08	0.8	10	19.9	26	43.2	18.8	28.6	22.5	19.2	10.8

09	3.1	16.2	21.4	18.9	40.4	30.2	26.1	17.6	21.0	5.2

11	0.0	3.4	7.6	8.9	80.2	41.3	23.3	25.4	9.8	0.2

12	10.4	17.6	29.6	24.1	18.3	28.9	28.3	21.6	15.6	5.7

13	26.4	29.1	23.3	13.2	8.1	29.6	19.1	16.7	19.9	14.7

14	10.6	20.7	20.3	26.5	21.8	20.4	26.1	29.0	15.0	9.5

15	18.5	21.0	22.6	22.2	15.7	16.7	24.7	23	22.1	13.6

16	23.4	25.0	22.1	19.2	10.3	23.3	21.2	22.2	17.4	15.9

* The material dimension is a socio-economic indicator based on education level, the employment/population ratio and the mean income. The social dimension takes into account the level of exclusion from a social network due to a separation, a divorce, widowhood, a single parenthood, or the fact of living alone.

^+ ^HR stands for health region

## Discussion

As reflected in the literature, the relationship between hospital admission for IHD and climate is probably a very complex one, modulated by several factors. Nonetheless, a strong relationship was observed in this study between IHD hospital admission rates and climatic conditions of the days preceding the hospitalization date. Delayed variables corresponding to ground snow, precipitation and daily mean temperature were among the first to be selected in the GAM model, confirming a cumulative effect of cold weather on IHD.

Some counter-intuitive findings also showed up. Cold temperatures resulted in a protective effect for women except for most Northern regions, pointing to possible links with protective behaviour (such as staying put at home during a cold spell), or the lower participation of women in the building or similar work sectors with compulsory outdoor exposure even during cold spells. In summer, for men 45-64 years old, a significant effect of hot temperatures was observed only in a few regions, whereas for women of the same age group the excess risk was recorded in most regions. In general the 45-64 age group was more at risk of IHD admission than the group of 65 years and over for both genders and in most regions. However, the older group was affected earlier in the season. Again behavioural factors are probably involved here, such as younger people having to work even in hot weather compared to retirees and seniors with activity limitations, as well as differential access to air conditioning, both already documented for the province [21,22].

A decline in the effects of meteorological variables on IHD daily admission rates was observed over the period of 1989-2006. This can partly be explained by the changes in surface air temperature over the period of our study. Studies have reported warming of the air temperature in Canada over the last few decades [23,24]. In Quebec, a warming of the surface air temperature is reported in the southern part of the province over the period of 1960 and 2005, taking place mostly after 1995 [25]. Also the increase trend during that period shows a gradient from South to North and West to East [25], which is consistent with our data. Also winters have been steadily warmer but summers have yet to become hotter for most regions [26].

The association between cold temperatures and IHD admission rates takes the same V or U shape as reported in the literature. However that shape was less common for summer models. The increase in daily hospitalizations with extreme temperatures was not observed in all cases (age groups, genders, health regions).

A decrease in hospital admissions was observed in all regions, for both genders and for both age groups, after an initial increase in the nineties. This is very consistent with the national trends of deaths and hospital admissions for heart diseases [1]. Tu et al. (2009) suggested that better control of lipid levels and associated coronary plaque stabilization through increasing rates of statin use in Canada could be significant factors in the decrease, with other medical treatment of risk factors also increasing and potentially involved in the trend [1]. Climate trends should be added as an explanatory variable of importance.

Regions with a significant excess risk also displayed high smoking prevalence and higher deprivation indexes (either material or social). A significant excess risk was also noted for the 65 years and over age group for some regions in which the smoking prevalence is moderate or low. This suggests that, in regions with high excess risk but moderate and low smoking prevalence, others risk factors not considered here are involved in the incidence of IHD. Diet, smoking and physical inactivity, considered modifiable risk factors, also contribute to the development of IHD. Only smoking could be addressed here, although the examination of the deprivation index allows for an assessment of the role of income, education, social support and similar important health determinants. Studies dealing with the impact of comorbidity on IHD, have reported hypertension, diabetes and obesity as known risk factors of cardiovascular diseases. Those risk factors increased in all age groups over the period of 1994-2004 and the highest increase was observed in the lower income groups [2]. Lee et al. (2009) conclude that the rising burden of those risk factors could result in future increases in cardiovascular diseases. They also reported that the smoking prevalence decreased over that period in all Canadian provinces. This can partly explain the drop in hospital admissions in all regions by the beginning of 2000.

The physiological processes that explain the effect of climate of ischemic heart disease are complex and not completely understood. Nonetheless, vitamin D is reported to be associated to the incidence of cardiovascular disease [27] and the mechanisms of its effect well documented [28]. Grimes, Hindle and Dyer [29] used the positive slope of the relationship between the concentration of blood cholesterol and latitude, the negative relationship between hours of sunshine per year and the death rate from coronary heart diseases (CHD) to suggest that the geographical variation of CHD is influenced by sunlight. Though we did not use sunlight exposure as an independent variable in our study, the separation of the seasons and the inclusion of the i^th ^day of the season in the models account for the seasonality of sunlight. Also, because of the known relationship between latitude and sunlight and thus, the cutaneous synthesis of vitamin D [30], the separation of data by health region can be construed as a form of stratification based on geographical coordinates. Figure 4 clearly shows a latitude effect on IHD hospital admission rate, with the annual IHD admission rate decreasing with the latitude.

The proportion of the population that is active is the same in all regions and cannot be considered as an explanatory variable for IHD hospitalisations, but the proportion of workers involved outdoors likely varies from region to region; unfortunately no data were available on this topic.

### Limitations of the study

Our study has some limitations. Because the health data were from existing administrative databases, patient history of heart diseases, personal habits such as smoking and comorbidity could not be taken into account. The limitations of data on smoking and the deprivation index allowed only for a qualitative analysis. Another limitation of our study is our inability to distinguish between new admissions and repeated ones, which could lead to increased rates of hospitalisation. Our study did not assess either the role of air pollution in the genesis of IHD episodes, although such a role seems significant [31-33].

## Conclusion

While these results should be considered preliminary, they nonetheless point out important messages for physicians throughout the country. Weather extremes do play a significant role in the genesis of acute and severe episodes of IHD, and the longer the heat wave or the cold spell, the more likely the public health impact. Several already vulnerable subgroups, based on a variety of indicators, seem to present more sensitivity to weather extremes, but adaptive behaviours can modify this excess risk. Adapted treatment regimes by physicians could probably help improve the score, although much more research at the individual level is warranted before reaching the stage of formal recommendations. However, older people and lower income groups could already benefit from supplementary medical attention in periods of meteorological extremes.

## Competing interests

The authors declare that they have no competing interests.

## Authors' contributions

All the authors contributed to the conception and design of the study, acquisition and interpretation of the data, and drafting and revising of the manuscript. All of the authors approved the final version submitted for publication.

## References

[B1] Tu JV Nardi L Fang J Liu J Khalid L Johansen H for the Canadian Cardiovascular Outcomes Research Team National trends in rates of death and hospital admissions related to acute myocardial infarction, heart failure and stroke, 1994-2004 CMAJ 2009 180 E118 125 19546444 10.1503/cmaj.081197 PMC2696549

[B2] Lee DS Chiu M Manuel DG Tu K Wang X Austin PC Mattern MY Mitiku TF Svenson LW Putnam W Flanagan WM Tu JV Canadian Cardiovascular Outcomes Research Team Trends in risk factors for cardiovascular disease in Canada: temporal, socio-demographic and geographic factors CMAJ 2009 181 E55 66 19620271 10.1503/cmaj.081629 PMC2717674

[B3] Dai S Bancej C Bienek A Walsh P Stewart P Weilgosz A Tracking heart disease and stroke in Canada 2009 Chronic Dis Can 2009 29 192 193

[B4] Lin S Luo M Walker RJ Liu X Hwang S-A Chinery R Extreme High Temperatures and Hospital Admissions for Respiratory and Cardiovascular Diseases Epidemiology 2009 20 738 746 10.1097/EDE.0b013e3181ad5522 19593155

[B5] Saez M Sunyer J Tobias A Ballester S Antó JM Ischemic heart disease mortality and weather temperature in Barcelona, Spain Eur J Public Health 2000 10 58 63 10.1093/eurpub/10.1.58

[B6] Chang CL Shipley M Marmot M Poulter N Lower ambient temperature was associated with an increased risk of hospitalization for stroke and acute myocardial infarction in young women J Clin Epidemiol 2004 57 749 757 10.1016/j.jclinepi.2003.10.016 15358404

[B7] Schnoor JL The IPCC fourth assessment Environ Sci Technol 2007 41 1503 10.1021/es072475x 17396625

[B8] Doyon B Belanger D Gosselin P The potential impact of climate change on annual and seasonal mortality for three cities in Quebec, Canada Int J Health Geogr 2008 7 23 10.1186/1476-072X-7-23 18498630 PMC2410102

[B9] Shao L Luo M Walker R Liu X Hwang S Robert C Impact of Hot Weather Conditions on Respiratory and Cardiovascular Hospital Admissions in New York City, USA Epidemiology 2008 19 S302 S303 10.1097/EDE.0b013e3181632c3d

[B10] Tiina MMk Raija J Jari J Terttu HH Ari P Aini B Sylvi S-K Maija L Juhani H Cold temperature and low humidity are associated with increased occurrence of respiratory tract infections Respir Med 2009 103 456 462 10.1016/j.rmed.2008.09.011 18977127

[B11] Friger M Yackerson N Bolotin A Kordysh E Meteorological Factors Influence on Hospitalization for Respiratory Diseases And Symptoms in the South Israel Epidemiology 2006 17 S423 S424 10.1097/00001648-200611001-01133

[B12] Schwartz J Samet JM Patz JA Hospital admissions for heart disease: the effects of temperature and humidity Epidemiology 2004 15 755 761 10.1097/01.ede.0000134875.15919.0f 15475726

[B13] Anderson RN Rosenberg HM Age standardisation of Death rates: Implementation of the year 2000 standard Natl Vital Stat Rep 1998 47 1 16 9796247

[B14] Pampalon R Hamel D Gamache P Raymond G A deprivation index for health planning in Canada Chronic Dis Can 2009 29 178 191 19804682

[B15] Morin A Receuil statistique sur la pauvreté et les inégalités socioéconomiques au Québec 2006 Québec: ISQ, MESS 134

[B16] Pampalon R Raymond G A deprivation index for health and welfare planning in Quebec Chronic Dis Can 2000 21 104 113 11082346

[B17] Puderer H Canada S Introducing the dissemination area for 2001 census: an uptade 2001 2000 Ottawa: Geography Working Paper Series 11

[B18] Environnement Canada Canada's National Climate Archive 2008 http://www.climate.weatheroffice.ec.gc.ca/climateData/canada_f.html

[B19] Hastie TJ Tibshirani RJ Generalized Additive Models 1990 1 New-York: Chapman & Hall

[B20] Eilers PHC Marx BD Flexible Smoothing with B-splines and Penalties Statist Sci 1996 11 89 120 10.1214/ss/1038425655

[B21] Bélanger D Gosselin P Valois P Abdous B Vagues de chaleur au Québec méridional: adaptations actuelles et suggestions d'adaptations futures 2006 Québec: Institut national de santé publique du Québec http://www.inspq.qc.ca/pdf/publications/538-VaguesChaleur_Quebec.pdf

[B22] Bélanger D Gosselin P Valois P Abdous B Vagues de froid au Québec méridional: adaptations actuelles et suggestions d'adaptations futures 2006 Québec: Institut national de santé publique du Québec http://www.inspq.qc.ca/pdf/publications/537-VaguesFroid_Quebec.pdf

[B23] Bonsal BR Zhang X Vincent LA Hogg WD Characteristics of daily and extreme temperatures over Canada J Clim 2001 14 1959 1976 10.1175/1520-0442(2001)014<1959:CODAET>2.0.CO;2

[B24] Zhang XB Vincent LA Hogg WD Niitsoo A Temperature and precipitation trends in Canada during the 20th century Atmosphere-Ocean 2000 38 395 429

[B25] Yagouti A Boulet G Vincent L Vescovi L Mekis E Observed changes in daily temperature and precipitation indices for southern Quebec, 1960-2005 Atmosphere-Ocean 2008 46 243 256 10.3137/ao.460204

[B26] Lemmen DS Warren FJ Lacroix J Bush E Lemmen Warren Vivre avec les changements climatiques au Canada: édition 2007 2008 Ottawa (Ontario): Gouvernement du Canada 448

[B27] Wang TJ Pencina MJ Booth SL Jacques PF Ingelsson E Lanier K Benjamin EJ D'Agostino RB Wolf M Vasan RS Vitamin D deficiency and risk of cardiovascular disease Circulation 2008 117 He 503 11 10.1161/CIRCULATIONAHA.107.706127 18180395 PMC2726624

[B28] Zittermann A Schleithoff SS Koerfer R Putting cardiovascular disease and vitamin D insufficiency into perspective Br J Nutr 2005 94 He 483 92 10.1079/BJN20051544 16197570

[B29] Grimes DS Hindle E Dyer T Sunlight, cholesterol and coronary heart disease QJM 1996 89 8 579 89 8935479 10.1093/qjmed/89.8.579

[B30] Webb AR Kline L Holick MF Influence of season and latitude on the cutaneous synthesis of vitamin D3: exposure to winter sunlight in Boston and Edmonton will not promote vitamin D3 synthesis in human skin J Clin Endocrinol Metab 1988 67 2 373 8 10.1210/jcem-67-2-373 2839537

[B31] Tagaris E Liao KJ Delucia AJ Deck L Amar P Russell AG Potential Impact of Climate Change on Air Pollution-Related Human Health Effects Environ Sci Technol 2009 43 4979 4988 10.1021/es803650w 19673295

[B32] Zanobetti A Schwartz J The Effect of Fine and Coarse Particulate Air Pollution on Mortality: A National Analysis Environ Health Perspect 2009 117 898 903 19590680 10.1289/ehp.0800108 PMC2702403

[B33] Cendon S Pereira LA Braga AL Conceicao GM Cury Junior A Romaldini H Lopes AC Saldiva PH Air pollution effects on myocardial infarction Rev Saude Publica 2006 40 414 419 10.1590/S0034-89102006000300008 16810364

